# Gigantol Improves Cholesterol Metabolism and Progesterone Biosynthesis in MA-10 Leydig Cells

**DOI:** 10.3390/cimb44010006

**Published:** 2021-12-23

**Authors:** Audrey Basque, Ha Tuyen Nguyen, Mohamed Touaibia, Luc J. Martin

**Affiliations:** 1 Biology Department, Université de Moncton, Moncton, NB E1A 3E9, Canada; eab7829@umoncton.ca (A.B.); ehn7853@umoncton.ca (H.T.N.); 2 Chemistry and Biochemistry Department, Université de Moncton, Moncton, NB E1A 3E9, Canada; mohamed.touaibia@umoncton.ca

**Keywords:** RNA-Seq, gigantol, Leydig cells, steroidogenesis, cholesterol metabolism

## Abstract

In aging males, androgen production by testicular Leydig cells decreases at a rate of approximately 1% per year. Phenolic compounds may enhance testosterone biosynthesis and delay the onset of male hypogonadism. Gigantol is a bibenzyl compound isolated from several types of orchids of the genus Dendrobium. This compound has various biological activities, including antioxidant activity. However, its capacity to regulate gene expression and steroid production in testicular Leydig cells has never been evaluated. We investigated the effect of gigantol on MA-10 Leydig cells’ gene expression using an RNA-Seq approach. To further investigate the structure-function relationship of the hydroxy-methoxyphenyl moiety of gigantol, experiments were also performed with ferulic acid and isoferulic acid. According to transcriptomic analysis, all genes coding for cholesterol biosynthesis-related enzymes are increased in response to gigantol treatment, resulting in increased lipid droplets accumulation. Moreover, treatments with 10 μM gigantol increased StAR protein levels and progesterone production from MA-10 Leydig cells. However, neither ferulic acid nor isoferulic acid influenced StAR protein synthesis and progesterone production in MA-10 Leydig cells. Thus, our findings indicate that gigantol improves cholesterol and steroid biosynthesis within testicular Leydig cells.

## 1. Introduction

In males, testosterone plays an essential role in testicular development, masculinization, and maintenance of sperm production. Reaching optimal levels in early adulthood, testosterone production by the Leydig cells of the testis declines with aging at a rate of about 1% per year [1]. However, several studies indicate that phenolic compounds can enhance testosterone synthesis and delay the decline in its production associated with aging and male hypogonadism [2].

Cholesterol constitutes the initial substrate of steroidogenesis in testicular Leydig cells. The two main sources of cholesterol are lipoproteins from the bloodstream and intracellular biosynthesis. In Leydig cells, the mevalonate pathway may contribute to supply more cholesterol during increased androgen biosynthesis. Androgen synthesis is initiated by the transport of cholesterol inside mitochondria through an import complex involving the steroidogenic acute regulatory (StAR) protein [3,4]. Cholesterol is then converted to pregnenolone by the enzyme CYP11A1. Pregnenolone is further metabolized into testosterone by other enzymes (HSD3B1, CYP17A1, HSD17B3) found in the endoplasmic reticulum. During aging, the decrease in StAR protein levels in Leydig cells leads to a disruption of cholesterol transport within mitochondria and a decrease in androgen production [5,6]. Flavonoids or their phenolic derivatives can delay this age-related decline in Leydig cell function by increasing the expression of *Star* and/or *Cyp11a1* genes [7]. In addition, flavonoids, such as quercetin, apigenin, and luteolin, enhance cAMP/PKA-dependent *Star* gene expression and steroidogenesis in Leydig cells by inhibiting cyclooxygenase-2 (COX2 or PTGS2) dependent signaling [7,8,9]. In aging males, the expression of PTGS2 in Leydig cells reduces *Star* gene expression and testosterone production [10].

Within Leydig cells, the expression of the *Star* gene is regulated by multiple transcription factors activated by the cAMP/PKA signaling pathway, including CREB1 and AP-1 family members [11,12]. CREB1 can be activated by polyphenolic compounds, such as quercetin [13]. Different phenolic compounds, such as resveratrol and curcumin, have been reported to inhibit the activity of AP-1 transcription factors cJUN and cFOS [14,15]. AP-1 members can activate or inhibit *Star* expression, depending on the nature of the homodimer or heterodimer being recruited to the *Star* promoter region [12]. Hence, phenolic compounds may improve androgen biosynthesis by increasing *Star* gene expression in Leydig cells.

Gigantol is a bibenzyl compound isolated from several types of orchids of the genus Dendrobium. This compound has various biological activities, including antioxidant activity [16], antispasmodic effect [17], antinociception [18], antiinflammation [18,19], and antiplatelet aggregation [20]. Related to cancer, gigantol has been reported to have antimigratory activity on lung cancer cells through a caveolin-1 dependent pathway [21,22] and anticancer activity in breast cancer cells by inhibition of Wnt/beta-catenin signaling [23]. It also inhibits epithelial to mesenchymal transition and induces anoikis in lung cancer cells by decreasing the expressions of N-cadherin, vimentin, slug, and phosphorylations of AKT and extracellular signal-regulated kinase (ERK) signaling pathways [24,25]. Others have reported that gigantol inhibits the proliferation of breast cancer cells by downregulation of the phosphoinositide 3-kinase/protein kinase B/mammalian target of rapamycin (PI3K/Akt/mTOR) signaling pathway [26]. The PI3K/Akt pathway is involved in a variety of biological processes, including cell growth, survival, and apoptosis, and also plays a role in cell metabolism, proliferation and migration [27,28]. Gigantol also has smooth muscle relaxant effects through the activation of the nitric oxide/cyclic GMP system [29] and the inhibition of calmodulin-sensitive phosphodiesterase [30]. However, the effects of gigantol on androgen biosynthesis have never been evaluated.

**Figure 1:**
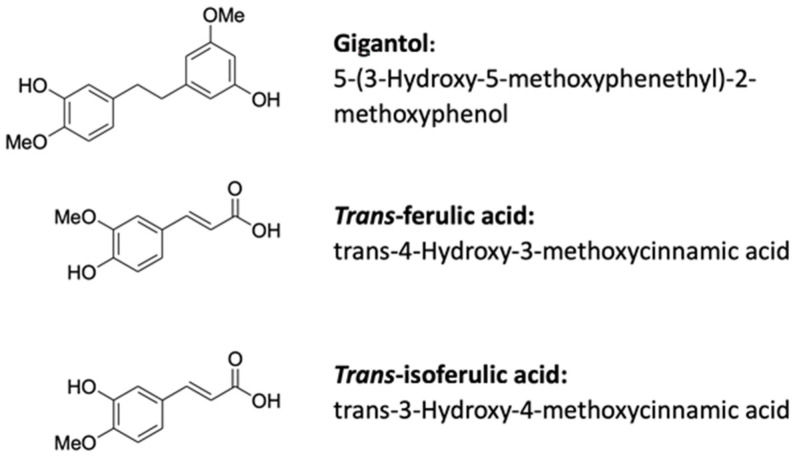
Schematic representation of gigantol (GOL), *trans*-ferulic acid (FA) and *trans*-isoferulic acid (IFA).

Since phenolic compounds, such as luteolin, can increase the steroidogenic capacity of Leydig cells in response to activation of the cAMP/PKA pathway, we investigated whether gigantol has the same ability. To further characterize the structure-function relationship of the methoxyphenyl moieties of gigantol, experiments were also performed with ferulic acid and isoferulic acid, also harboring hydroxy-methoxyphenyl moiety (Figure 1). Having a common substructure with gigantol, ferulic acid can be found in several plants [31]. The isomer of ferulic acid, isoferulic acid, which is much less common in plants, is widely used in traditional Chinese herbal medicines. It is found in *Cimicifuga heracleifolia*, which is used for the treatment of diseases linked to the generation of reactive oxygen species often associated with the deregulation of the antioxidant defense mechanisms [32,33]. Ferulic acid and isoferulic acid belong to the phenolic subfamily of hydroxycinnamic acids. Ferulic acid has been reported to improve sperm parameters of diabetic rats [34], as well as sperm viability and motility of infertile men [35]. We investigated the effect of gigantol on MA-10 Leydig cells’ gene expression using an RNA-Seq approach. Our findings indicate that gigantol can improve cholesterol and steroid biosynthesis within testicular Leydig cells.

## 2. Materials and Methods

### 2.1. Chemicals

Gigantol (GOL), *trans*-ferulic acid (FA), *trans*-isoferulic acid (IFA), and forskolin (FSK) were purchased from Sigma-Aldrich Canada (Oakville, ON, Canada).

### 2.2. Cell Culture and Treatments

Mouse MA-10 Leydig cells were obtained from American Type Culture Collection (Manassas, VA, USA) and cultured as described previously [36]. After platting and incubation for 48 h, MA-10 cells were treated for 4 h with increasing concentrations (1, 10, 100 μM) of gigantol (GOL), ferulic acid (FA), or isoferulic acid (IFA) in the absence or presence of forskolin (FSK) at 10 μM in DMEM/F12 without serum and antibiotics before being harvested for RNA or for protein extractions.

### 2.3. Cell Viability

Mouse MA-10 Leydig cells were plated in 96 wells and incubated for 72 h before cell viability assays. Following treatments and incubations, cell viability was measured using the crystal violet cell staining assay. Briefly, cultured cells were fixed using 10% formalin, stained with 0.25% Crystal Violet, and washed with distilled water. Retained coloration was then solubilized in 33% glacial acetic acid and quantified by absorbance at 595 nm using a multimode microplate reader (Varioskan, Thermo Scientific, Waltham, MA, USA). Experiments were repeated for four biological replicates.

### 2.4. RNA Extraction and RNA Sequencing Analyses

After treatments of MA-10 Leydig cells, total RNA was isolated with E.Z.N.A. Total RNA kit (Omega Bio-Tek, Inc., Norcross, GA, USA). RNA quality was determined by electrophoresis and characterization of the 18S and 28S ribosomal RNA bands. Libraries were prepared using the NEBNext Stranded mRNA kit (New England BioLabs, Whitby, ON, Canada) for cDNA synthesis and polyA enrichment. Quality and quantification of libraries were evaluated using the Agilent 2100 Bioanalyzer (Agilent Technologies, Mississauga, ON, Canada) and KAPA SYBR^®^ FAST qPCR Kit (KAPA Bio Systems, Wilmington, MA, USA), respectively. Transcriptome sequencing was performed on an Illumina HiSeq 4000 in paired reads layout with approximately 35.5 million reads per sample and 100 bp of sequencing length. Libraries and next generation sequencing were completed by the McGill University and Genome Quebec Innovation Center (Montreal, QC, Canada). Raw reads in FASTQ format can be accessed on NCBI-SRA at the BioProject ID PRJNA783636. RNA-Seq analyses were performed using the Galaxy platform from Compute Canada (https://www.genap.ca/accessed on 29 June 2021). Paired-end reads were aligned to mouse reference genome (mm10) and identified according to the GENCODE release M10 (GRCm38.p4) comprehensive gene annotation using the RNA-STAR read mapper (version 2.7.5 b) [37]. Assemblies and quantifications of transcripts were performed on BAM files using the tool featureCounts with settings allowing reads to contribute to multiple features (version 1.6.2) [38]. Differential gene expression was assessed on normalized gene counts using DESeq2 (version 2.11.40.2) for parametric fit type [39]. Results were filtered for adjusted *p* values lower than 0.05 for differential expression in response to gigantol treatment.

### 2.5. Pathways and Networks Analyses

Differentially expressed genes were analyzed using the gProfiler platform (version e104_eg51_p15_3922dba, database updated on 30 June 2021) for functional enrichment of biological process and pathway annotations using the g:SCS significance threshold [40,41]. Results for genes upregulated for steroid biosynthetic process were visualized using GeneMANIA (version 3.6.0) [42]. From the total gene list of differentially expressed genes, we performed a gene set enrichment analysis using the GSEA software (version 4.1.0) from the Broad Institute [43]. A ranking of the differential expression results was determined by score calculation based on fold changes and *p*-values of DESeq2 results (sign(logFC)*-log10(p value)) and compared to mouse gene ontology annotations (cell component, molecular function and biological process), released 1 March 2021 (http://download.baderlab.org/EM_Genesets/ accessed on 28 September 2021), using a modified Kolmogorov-Smirnov statistic.

### 2.6. Quantification of Lipid Droplets

After platting MA-10 Leydig cells in 96-well plates and incubation for 72 h, cells were treated and lipid droplets were quantified using Nile Red. Briefly, cells were washed two times with phosphate buffered saline, followed by staining with a solution containing 10 mg/L Nile Red and 100 mg/L Hoechst 33342. After 15 min of incubation at room temperature, cells were washed three times with phosphate buffered saline and submerged in 50 μL of phosphate buffered saline before reading using a Varioskan microplate reader (Thermo Scientific, Waltham, MA, USA). Conditions for detection of fluorescence were 361 nm excitation/497 nm emission for Hoechst and 515 nm excitation/585 nm emission for Nile Red. Quantification of lipid droplets was reported as the ratio of Nile Red fluorescence on Hoechst fluorescence.

### 2.7. Western Blot Assays

Following treatments of MA-10 tumor Leydig cells, total protein extractions were obtained using the RIPA lysis buffer. Protein concentrations were determined using the Bradford method [44]. Thirty micrograms of total protein extracts were separated by SDS–polyacrylamide gel electrophoresis and transferred onto a PVDF membrane. Membranes were blocked with 5% non-fat dry milk in TBST buffer (TBS buffer containing 0.05% Tween 20) for 1 h at room temperature, followed by an incubation with a specific primary antibody overnight at 4 °C. Target proteins were detected using a monoclonal anti-StAR (1:1000, Cat.: 8449, Cell Signaling Technology, Danvers, MA, USA), a monoclonal anti-CREB (1:1000, Cat.: 4820, Cell Signaling Technology, Danvers, MA, USA), a polyclonal anti-IDI1 (1:2000, Cat.: 11166-2-AP, ProteinTech Group, Inc., Rosemont, IL, USA), a polyclonal anti-LSS (1:1000, Cat.: 13715-1-AP, ProteinTech Group, Inc., Rosemont, IL, USA), a polyclonal anti-NSDHL (1:2000, Cat.: 15111-1-AP, ProteinTech Group, Inc., Rosemont, IL, USA), a polyclonal anti-MVD (1:1000, Cat.: 15331-1-AP, ProteinTech Group, Inc., Rosemont, IL, USA), or a polyclonal anti-MVK (1:2000, Cat.: 12228-1-AP, ProteinTech Group, Inc., Rosemont, IL, USA) antibody. The membranes were washed three times in TBST buffer for 5 min, followed by an incubation with horseradish peroxidase-conjugated secondary antibody (1:5000, New England BioLabs, Whitby, ON, CAN) for 1 h. Tubulin-β (TUBB) was detected for total protein extracts control purposes using monoclonal anti-TUBB (1:100,000, Cat.: 66240-1-IG, ProteinTech Group, Inc., Rosemont, IL, USA). Final revelation was performed using the Luminata Forte chemiluminescence detection system (Millipore, Billerica, MA, USA). Images were taken using the ChemiDoc MP imaging system (Bio-Rad, Hercules, CA, USA).

### 2.8. Plasmid Constructs and Transfection Assays

The −902 to +17 bp mouse *Star* and the −1003 to +45 bp mouse *Fdx1* luciferase promoter/reporter constructs were described previously [45,46]. The p3xAP1-luc reporter plasmid (40342) was obtained from Addgene (Cambridge, MA, UAS), and the pCREB-luc reporter plasmid was purchased from Signosis Inc. (Santa Clara, CA, USA). MA-10 cells were transfected using polyethylenimine according to the previously described method [36]. Each experiment was performed as technical triplicates.

### 2.9. Progesterone Quantification Assays

ELISAs for progesterone quantification were performed as recommended by the manufacturer (DRG International Inc., Springfield, NJ, USA). Following treatments, the culture medium was transferred to 1.5 mL tubes and stored at -80 °C until ELISAs were performed.

### 2.10. Statistical Analyses

Significant expression differences between control and treated conditions were characterized using a one-way ANOVA followed by a Holm-Sidak’s multiple comparison test. For all statistical analyses, *p* < 0.05 was considered significant. Statistical analyses were performed using the GraphPad Prism 9.2.0 software package (GraphPad Software Inc., La Jolla, CA, USA).

## 3. Results

### 3.1. Gigantol Increases the Expression of Genes Involved in Cholesterol and Steroid Metabolic Processes

**Figure 2:**
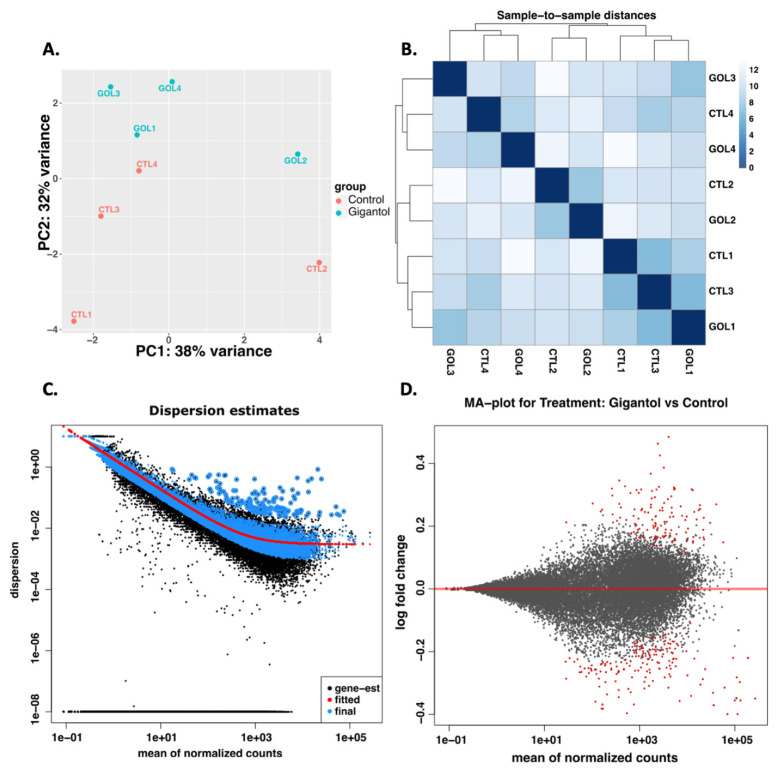
Overview of RNA-Seq data from MA-10 Leydig cells treated with gigantol 10 μM for 4 h. (**A**) The principal component analysis results showing that samples from gigantol treatment are clustered compared to non-treated samples. (**B**) Sample-to-sample distance heatmap performed on transcripts identified in non-treated and gigantol-treated MA-10 Leydig cells from four independent biological replicates. (**C**) The dispersion estimate plot shows the gene-wise estimates (black), the fitted values (red), and the final maximum a posteriori estimates used in testing (blue), where the black points circled in blue are detected as dispersion outliers. (**D**) The MA plot of RNA-Seq data compares gigantol treated to control samples. The *x*-axis shows mean expression for each gene, and the *y*-axis shows log2 fold change (gigantol treated versus control), as estimated in DESeq2.

Following MA-10 tumor Leydig cells’ treatment with gigantol at 10 μM for 4 h, RNA extractions were performed, and the transcriptome was characterized by RNA-Seq analyses. In response to gigantol, the expressions of 83 genes were upregulated and that of 120 genes were downregulated (*p*_adj_ < 0.05). As presented in the principal component analysis (Figure 2A), RNA-Seq data variation was more important between samples of the same group than between treatment conditions. Hence, sample-to-sample distances of RNA-Seq data (Figure 2B) indicated that there was no grouping of gigantol treated samples compared to DMSO (CTL group). The graphical representation of dispersion estimates (Figure 2C) shows that final gene estimates are mostly grouped toward the fitted estimate values and that few genes’ estimates can be considered as outliers. According to MA (log ratio to mean average)-plot of differentially expressed genes (Figure 2D), gigantol treatment has a minimal effect on gene regulation with log2 fold changes of 0.48 or less.

**Figure 3:**
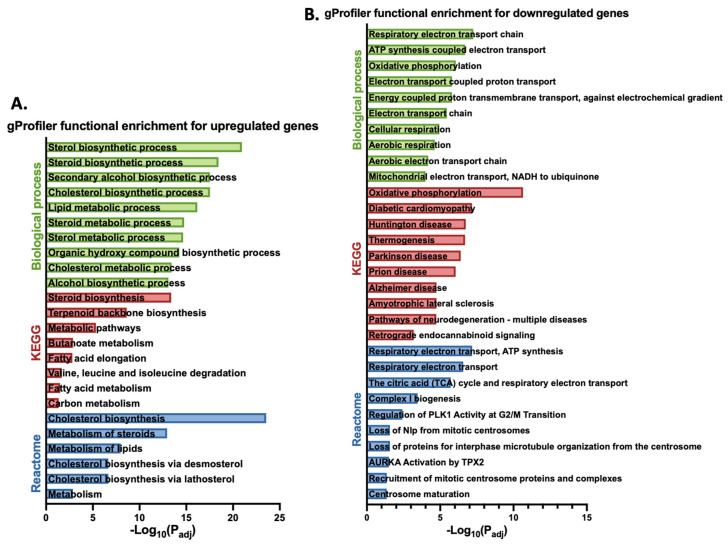
Functional enrichment for genes being upregulated (**A**) or downregulated (**B**) by gigantol in MA-10 Leydig cells according to the gProfiler analysis. Only the top 10 biological processes, KEGG pathways, and Reactome pathways are presented.

**Figure 4:**
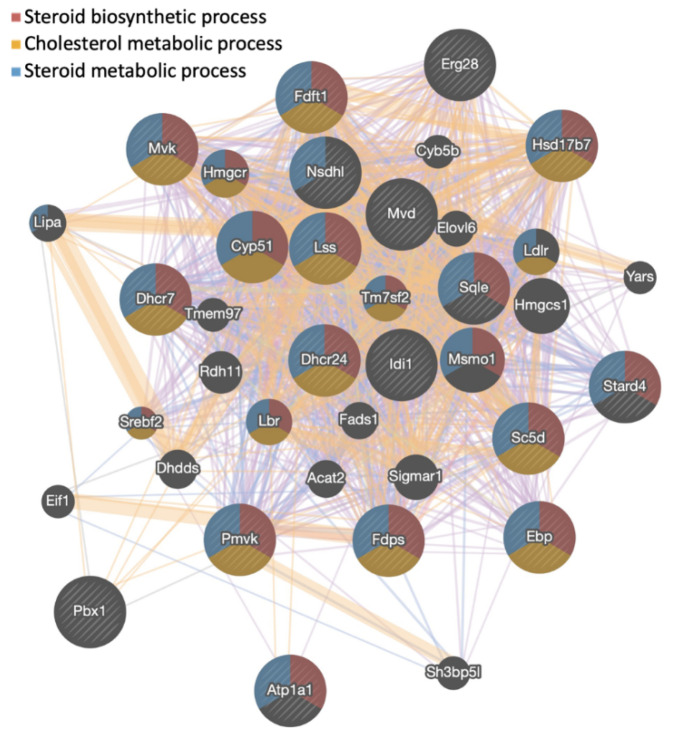
Network analysis of genes being upregulated by gigantol in MA-10 Leydig cells using the GeneMania function prediction tool.

**Figure 5:**
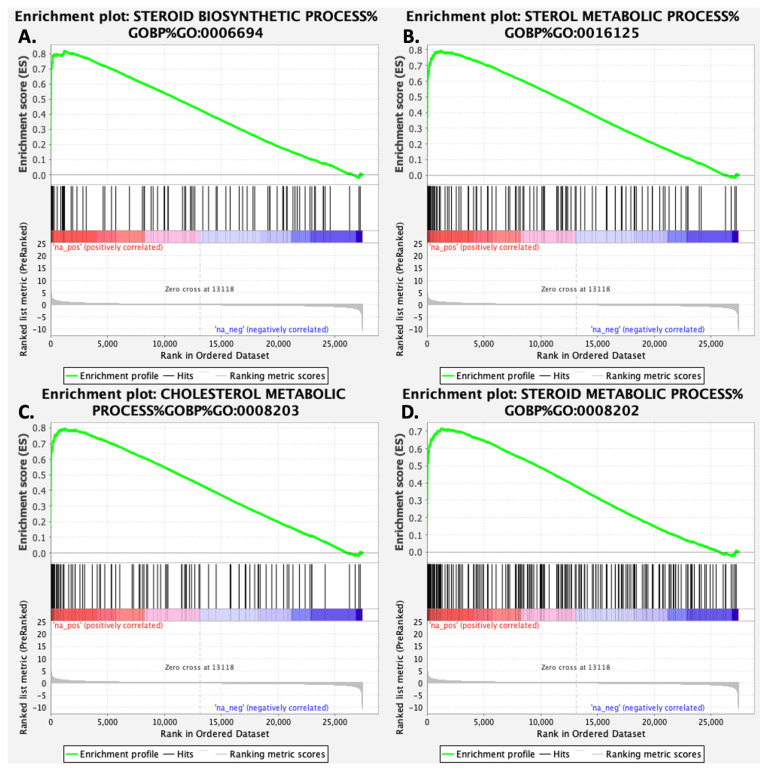
Enrichment plots from gene set enrichment analysis (GSEA) of genes modulated by gigantol in MA-10 Leydig cells. (**A**) Steroid biosynthetic process, (**B**) sterol metabolic process, (**C**) cholesterol metabolic process, and (**D**) steroid metabolic process are important biological processes for Leydig cells’ normal functions.

Following an analysis of genes with increased expression in response to gigantol using the gProfiler platform, differentially expressed genes are enriched for biological processes and pathways related to cholesterol biosynthesis, steroid biosynthesis, and steroid metabolism (Figure 3A). Genes with decreased expression in response to gigantol were rather enriched for biological processes and pathways related to electron transport chain, oxidative phosphorylation, and the citric acid cycle (Figure 3B). Genes being upregulated by gigantol were also analyzed using the GeneMANIA function prediction tool. As indicated in Figure 4, gigantol upregulated genes having links to steroid biosynthesis, as well as cholesterol and steroid metabolism. Further supporting these functional associations, RNA-Seq data was analyzed using GSEA (gene set enrichment analysis) with the mouse gene ontology annotations. Indeed, genes being upregulated by gigantol are enriched for steroid biosynthetic and metabolic processes, as well as for sterol and cholesterol metabolic processes (Table 1 and Figure 5) (FDR q-value < 0.25). Genes being downregulated by gigantol are rather linked to base pairing, translation elongation, mRNA splice site selection, ATP synthesis coupled electron transport, and oxidative phosphorylation (FDR q-value < 0.25).

**Figure 6:**
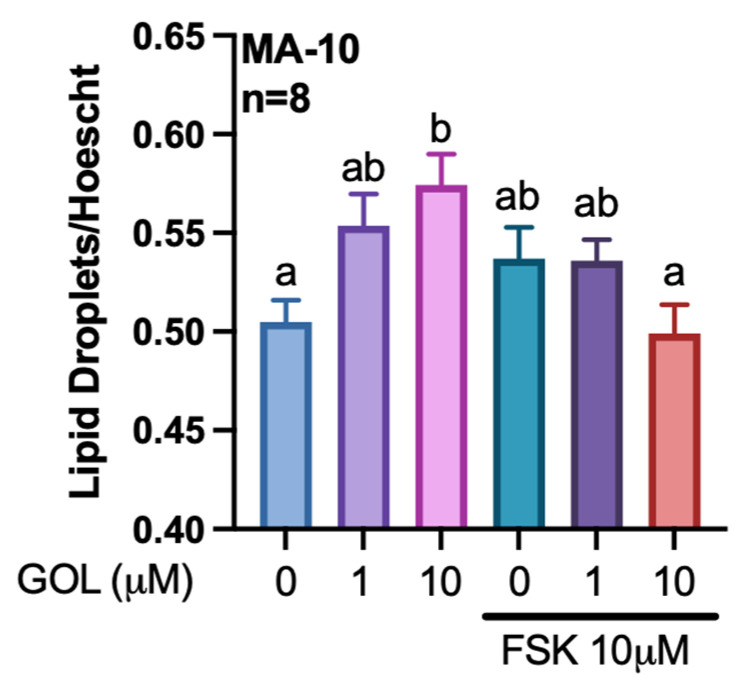
Changes in the accumulation of lipid droplets in response to gigantol treatment of MA-10 Leydig cells. Following treatments of MA-10 Leydig cells with gigantol (1, 10 μM) in the presence or absence of 10 μM forskolin (FSK) for 4 h, lipid droplets were quantified using Nile Red staining. Results are normalized using Hoescht DNA staining. A two-way ANOVA followed by a Holm-Sidak multiple comparison test was used to analyze data. Different letters indicate significant differences (*p* < 0.05).

In Leydig cells, newly synthesized cholesterol can be accumulated in lipid droplets. Thus, the abundance of lipid droplets was quantified in gigantol-treated MA-10 Leydig cells by Nile Red staining. Consistent with an increase in the expression of genes involved in cholesterol biosynthesis, gigantol at 10 μM increases the abundance of lipid droplets formed in MA-10 cells (Figure 6). However, this accumulation of lipid droplets is not increased by co-treatments with forskolin (FSK), an activator of adenylate cyclase and of the cAMP/PKA pathway.

### 3.2. Gigantol Increases STAR mRNA Expression and Protein Levels

**Figure 7:**
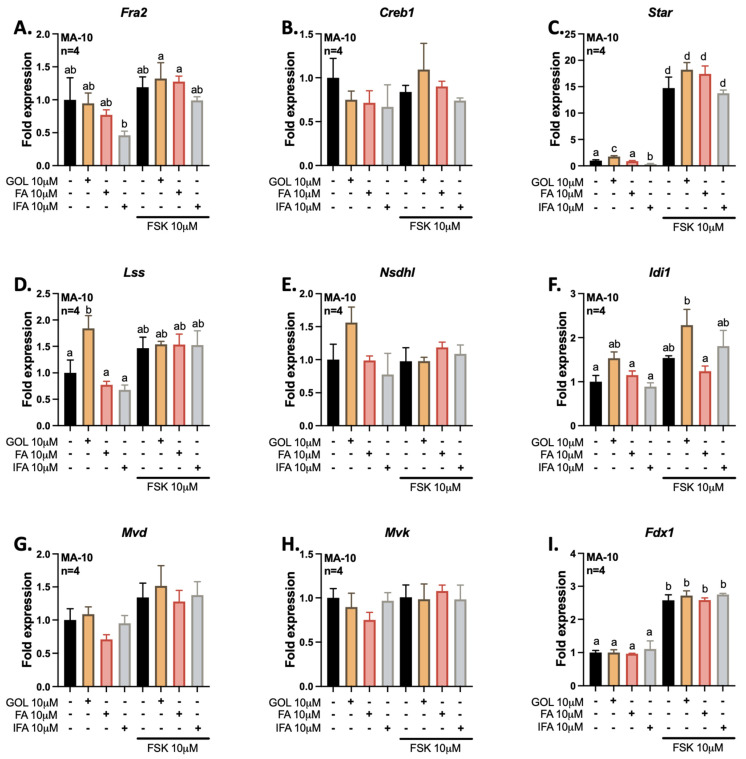
Evaluation of gene expression by quantitative real-time PCR for genes related to the regulation of steroidogenesis and cholesterol biosynthesis. Following treatments of MA-10 Leydig cells with 10 μM gigantol (GOL), ferulic acid (FA), or isoferulic acid (IFA) in the presence or absence of 10 μM forskolin (FSK) for 4 h, total RNAs were extracted, followed by reverse transcription and quantitative real-time PCR assays for (**A**) *Fra2*, (**B**) *Creb1*, (**C**) *Star*, (**D**) *Lss*, (**E**) *Nsdhl*, (**F**) *Idi1*, (**G**) *Mvd*, (**H**) *Mvk* and (**I**) *Fdx1* gene expressions. Results are normalized using *Rpl19* reference gene expression and presented as fold expression relative to control (nontreated cells, DMSO only) (±SEM). A two-way ANOVA followed by a Holm-Sidak multiple comparisons test was used to analyze data. Different letters indicate significant differences (*p* < 0.05).

Then, we evaluated the abilities of gigantol, as well as related ferulic and isoferulic acids, to modulate the expressions of genes related to steroidogenesis and cholesterol metabolism (Figure 7). We performed co-treatments with the adenylate cyclase activator FSK to determine whether these phenolic compounds can modulate the upregulation of these genes via activation of the cAMP/PKA signaling pathway. The transcription factors Fra2 and Creb1 have been shown to be upregulated by gigantol by RNA-Seq; however, the results could not be confirmed by qPCR (Figure 7A,B). As reported previously [45], *Star* expression is increased in response to activation of the cAMP/PKA pathway. However, only gigantol can increase on *Star* expression in the absence of cAMP/PKA pathway activation (Figure 7C). Supporting RNA-Seq data for genes related to cholesterol metabolism, the expression of *Lss* is increased in response to gigantol (Figure 7D). Although non-significant, the expression of *Nsdhl* seems to be improved by gigantol (Figure 7E). In addition, the expression of *Idi1* in response to activation of the cAMP/PKA pathway is improved by gigantol (Figure 7F). However, the upregulations of *Mvd*, *Mvk*, and *Fdx1* by gigantol could not be confirmed by qPCR assays (Figure 7G–I).

**Figure 8:**
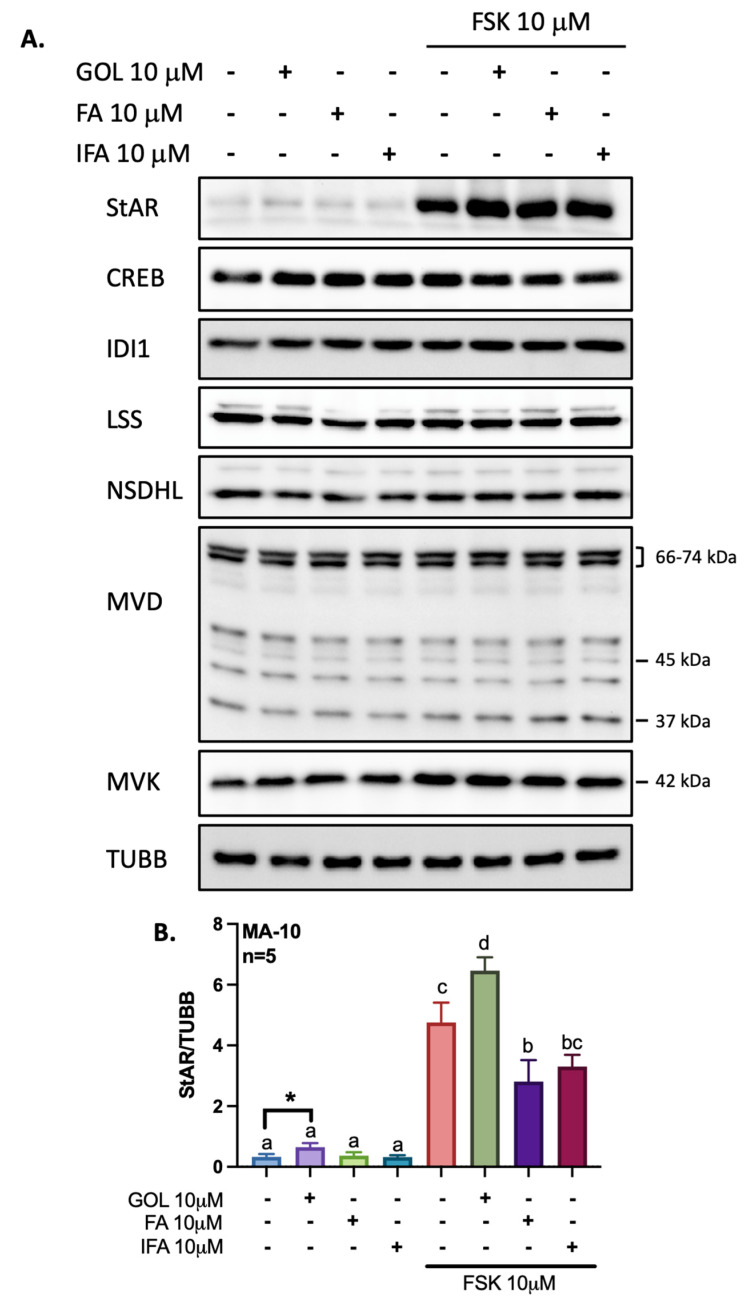
Total protein levels for genes related to regulation of steroidogenesis and cholesterol biosynthesis. (**A**) Following treatments of MA-10 Leydig cells with 10 μM gigantol (GOL), ferulic acid (FA), or isoferulic acid (IFA) in the presence or absence of 10 μM forskolin (FSK) for 4 h, total proteins were extracted, followed by quantification using Western blot assays. Results are normalized using Tubulin β (TUBB). (**B**) Densitometry ratios of StAR to TUBB are presented. A two-way ANOVA followed by a Tukey’s multiple comparison test was used to analyze data on densitometries. Different letters indicate significant differences (*p* < 0.05). The asterisk (*) indicates a statistically significant difference using a paired student *t*-test (*p* = 0.0081).

Other than influencing gene expression, the phenolic compounds being investigated could also modulate the levels of proteins regulating steroidogenesis and cholesterol biosynthesis in Leydig cells. Hence, we performed Western blots analyses on total protein extracts from MA-10 Leydig cells being treated with gigantol, ferulic acid, or isoferulic acid in the presence or absence of FSK (Figure 8). Interestingly, gigantol alone consistently increases StAR protein levels in MA-10 Leydig cells as confirmed using a paired student *T-*test (Figure 8B). Only StAR protein level is increased in response to FSK, and only co-treatment with gigantol improves FSK-dependent StAR protein accumulation (Figure 8B). Protein levels for CREB1, IDI1, LSS, NSDHL, MVD, and MVK are not altered by treatments of MA-10 cells (Figure 8A). While multiple bands were obtained with the MVD antibody, none were modulated in response to treatments of MA-10 cells. Although increased at the mRNA level in response to FSK (Figure 7I), FDX1 protein levels could not be assessed because of the lack of available antibody.

### 3.3. Gigantol Improves Progesterone Production in MA-10 Leydig Cells

**Figure 9:**
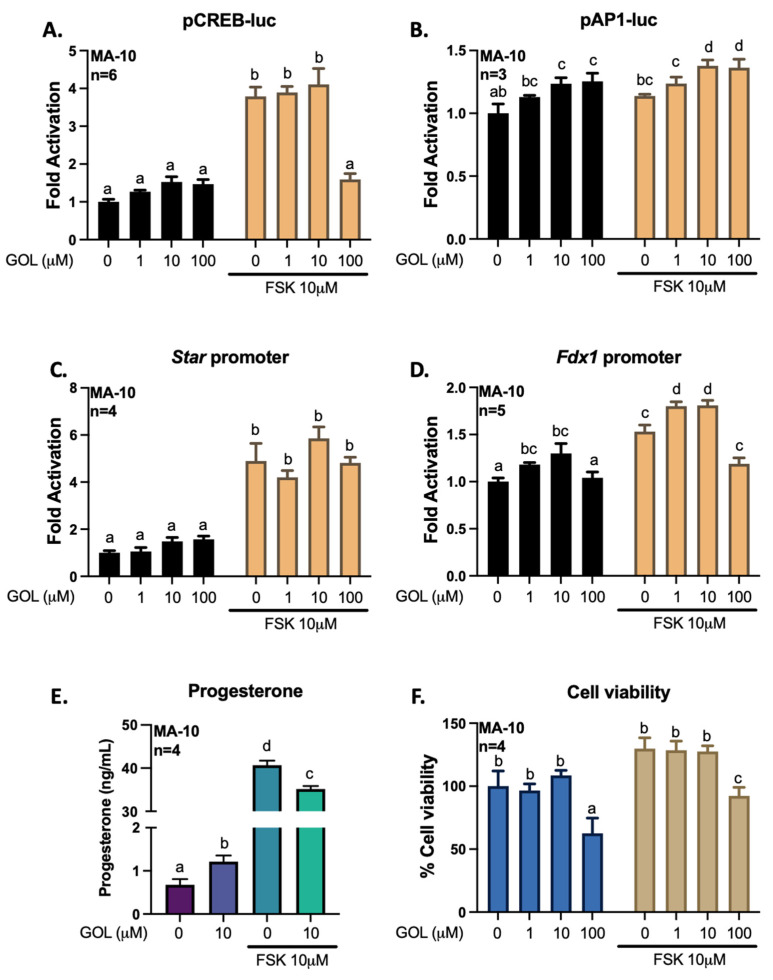
Effects of increasing levels of gigantol (GOL) on the activities of pCREB-luc (**A**), pAP1-luc (**B**), *Star* promoter (**C**), or *Fdx1* promoter (**D**). MA-10 Leydig cells were transfected with a luciferase plasmid reporter harboring 4 consensus CREB regulatory elements, 3 consensus AP1 regulatory elements, the −902 to +17 bp mouse *Star* promoter, or the −1003 to +45 bp *Fdx1* promoter, upstream of the gene for *Firefly* luciferase. Cells were treated for 4 h with increasing doses of gigantol (1, 10 or 100 μM) with or without the adenylate cyclase activator forskolin (FSK) at 10 μM. Results are shown as Fold Activation over control (DMSO only) (±SEM). Progesterone was quantified by ELISA from the cell culture medium (**E**), and cell viability (**F**) was determined using the Crystal Violet method. A two-way ANOVA was used to analyze data according to concentrations of gigantol and stimulation with FSK, followed by Holm-Sidak’s multiple comparison test. A different letter denotes a significant difference (*p* < 0.05).

**Figure 10:**
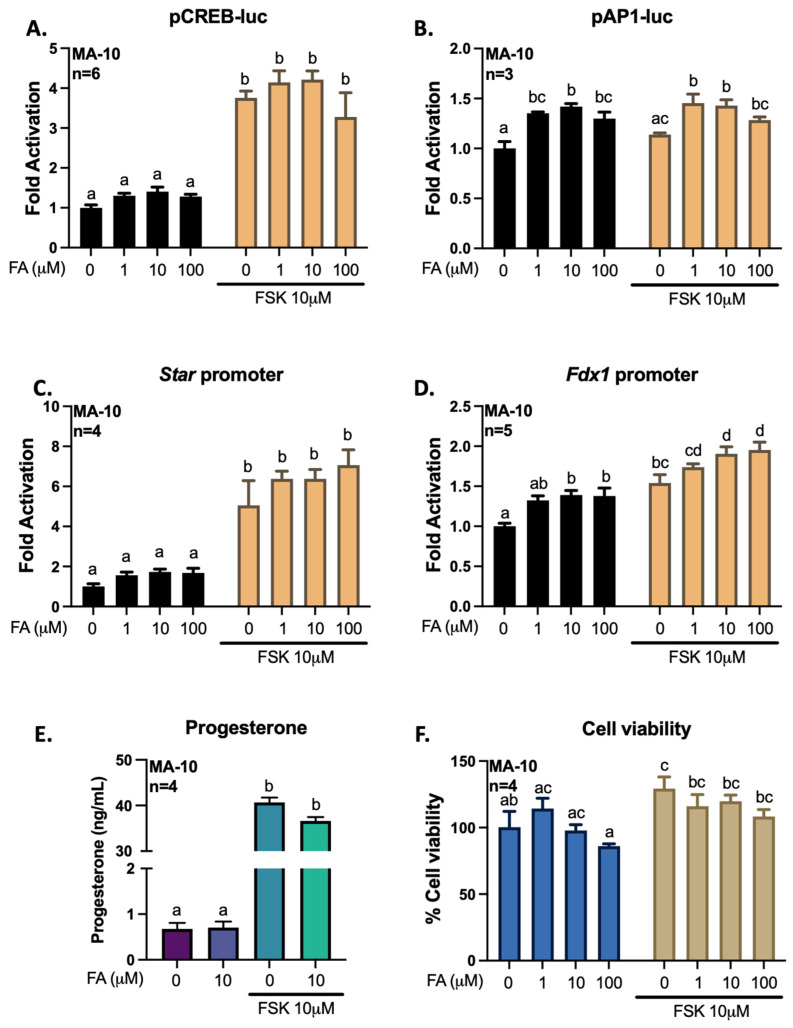
Effects of increasing levels of ferulic acid (FA) on the activities of pCREB-luc (**A**), pAP1-luc (**B**), *Star* promoter (**C**), or *Fdx1* promoter (**D**). MA-10 Leydig cells were transfected with a luciferase plasmid reporter harboring 4 consensus CREB regulatory elements, 3 consensus AP1 regulatory elements, the −902 to +17 bp mouse *Star* promoter, or the −1003 to +45 bp *Fdx1* promoter, upstream of the gene for *Firefly* luciferase. Cells were treated for 4 h with increasing doses of ferulic acid (1, 10, or 100 μM) with or without the adenylate cyclase activator forskolin (FSK) at 10 μM. Results are shown as Fold Activation over control (DMSO only) (±SEM). Progesterone was quantified by ELISA from the cell culture medium (**E**), and cell viability (**F**) was determined using the Crystal Violet method. A two-way ANOVA was used to analyze data according to concentrations of ferulic acid and stimulation with FSK, followed by Holm-Sidak’s multiple comparison test. A different letter denotes a significant difference (*p* < 0.05).

**Figure 11:**
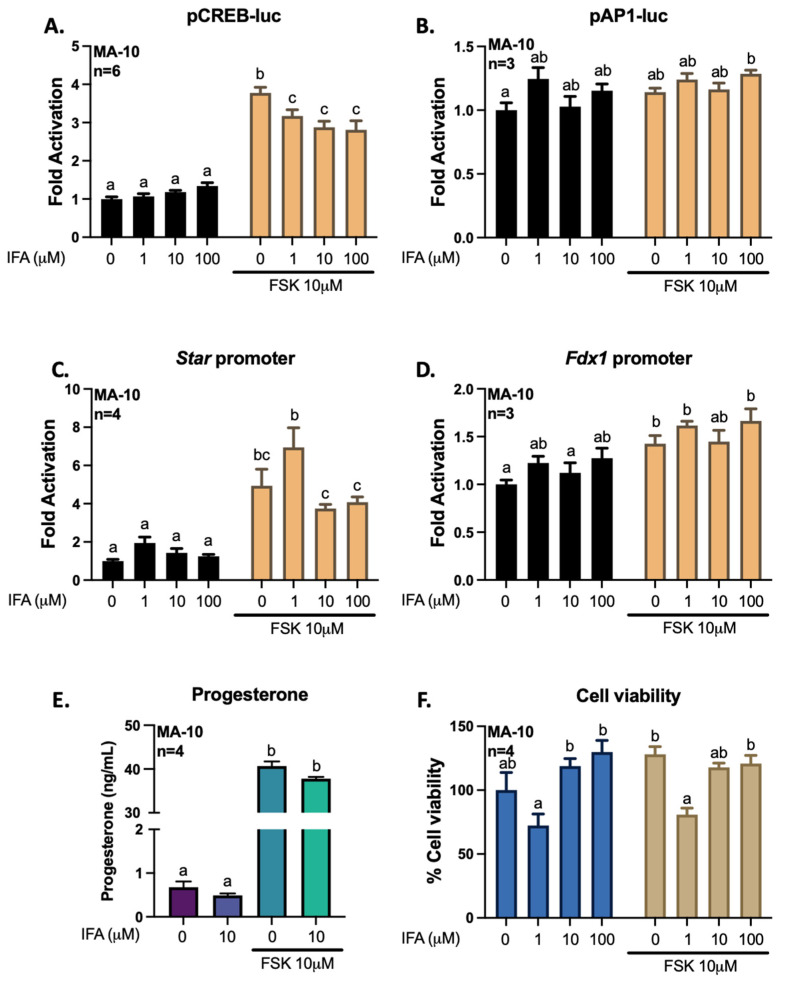
Effects of increasing levels of isoferulic acid (IFA) on the activities of pCREB-luc (**A**), pAP1-luc (**B**), *Star* promoter (**C**), or *Fdx1* promoter (**D**). MA-10 Leydig cells were transfected with a luciferase plasmid reporter harboring 4 consensus CREB regulatory elements, 3 consensus AP1 regulatory elements, the −902 to +17 bp mouse *Star* promoter, or the −1003 to +45 bp *Fdx1* promoter, upstream of the gene for *Firefly* luciferase. Cells were treated for 4 h with increasing doses of isoferulic acid (1, 10, or 100 μM) with or without the adenylate cyclase activator forskolin (FSK) at 10 μM. Results are shown as Fold Activation over control (DMSO only) (±SEM). Progesterone was quantified by ELISA from the cell culture medium (**E**), and cell viability (**F**) was determined using the Crystal Violet method. A two-way ANOVA was used to analyze data according to concentrations of isoferulic acid and stimulation with FSK, followed by Holm Sidak’s multiple comparison test. A different letter denotes a significant difference (*p* < 0.05).

To better define how gigantol, ferulic, and isoferulic acids can regulate the transcription of genes related to steroidogenesis, we transfected MA-10 cells with promoter constructs coupled to a *Firefly luciferase* reporter gene. The CREB-luc reporter plasmid harboring consensus regulatory elements for CREB1 confirms the activation of the cAMP/PKA pathway by FSK. The AP1-luc reported plasmid harboring consensus regulatory elements for transcription factors members of the AP-1 family, such as FRA2, confirms the activation of the AP-1 transcription factors. Thus, following transfection of a promoter-reporter constructs containing regulatory elements for CREB, regulatory elements for AP1, the −902 to +17 bp promoter region of *Star* or the −1003 to +45 bp promoter region of *Fdx1*, MA-10 cells were treated with gigantol, ferulic, or isoferulic acid in the absence or presence of FSK for 4 h (Figure 9, Figure 10 and Figure 11).

Increasing concentrations of gigantol (1–100 μM) has no stimulatory effect on basal or FSK-dependent activation of CREB activity and *Star* promoter-reporter constructs (Figure 9A,C). However, gigantol significantly increases the transcriptional activity of AP-1 members at concentrations of 10 and 100 μM (Figure 9B). In addition, gigantol at 1 and 10 μM increases basal and cAMP/PKA-dependent *Fdx1* promoter activity (Figure 9D). In Leydig cells, FDX1 promotes steroid biosynthesis through electron transfer to the rate-limiting steroidogenic enzyme, CYP11A1. Indeed, 10 μM gigantol increases progesterone synthesis by MA-10 Leydig cells (Figure 9E). However, such effect could not be observed in the presence of FSK. Inhibition of the activity of CREB in response to high levels of gigantol may be attributed to decreased cell viability under such treatments’ conditions (Figure 9F).

Treatments with ferulic acid have no significant effects on CREB transcriptional activity and *Star* promoter activation (Figure 10A,C). However, ferulic acid (1–100 μM) increases the activity of AP-1 members, as well as the basal and cAMP/PKA-dependent *Fdx1* promoter activity (Figure 10B,D). Nevertheless, ferulic acid has no effect on progesterone production and on the viability of MA-10 Leydig cells (Figure 10E,F).

As opposed to ferulic acid, treatments with isoferulic acid inhibit FSK-dependent increases in CREB activity and *Star* promoter activation (Figure 11A,C). Moreover, the activity of AP-1 factors and of the *Fdx1* promoter are not altered by isoferulic acid (Figure 11B,D). Thus, isoferulic acid has no effect on progesterone production in MA-10 Leydig cells (Figure 11E). Although treatment with a 1 μM isoferulic acid leads to a non-significant increase in basal and cAMP/PKA-dependent activation of the *Star* promoter, this effect could be diminished by the reduction in cell viability under these same treatment conditions (Figure 11C,F).

## 4. Discussion

According to transcriptomic analyses of MA-10 Leydig cells, gigantol increases the expression of genes involved in cholesterol and steroid biosynthesis/metabolic processes. Gigantol also increases the activity of transcription factors members of the AP-1 family and promotes basal and cAMP/PKA-dependent activation of StAR protein synthesis. As a result of its actions on steroidogenic gene expression, gigantol improves the production of progesterone from MA-10 Leydig cells. However, such improvement is not dependent on activation of the cAMP/PKA pathway.

In our RNA-Seq experiments, the stimulatory effects of gigantol on the expression of genes involved in cholesterol and steroid biosynthesis may be attributed to its antioxidant properties [47,48]. The accumulation of free radicals, particularly reactive oxygen species, in cells in the absence of antioxidants leads to the development of oxidative stress [49]. The severity of oxidative stress decreases cell functions, which can be attributed to increased oxidative damage to functional proteins [50]. During male aging, such a state of oxidative stress in the Leydig cells of the testis can reduce the synthesis of cholesterol and testosterone [51]. In general, oxidative stress reduces the endogenous levels of enzymatic and non-enzymatic antioxidants in Leydig cells [52]. Thus, phenolic compounds, including gigantol, may improve Leydig cell functions, such as cholesterol synthesis and testosterone production, by enhancing antioxidant defense mechanisms. Indeed, other polyphenolic compounds, such as luteolin and quercetin, have been reported to activate the expressions of glutathione S-transferases, *Gsta1* and *Gstt2*, and decrease the levels of reduced glutathione in LC540 tumor Leydig cells [53]. However, the implication of gigantol in the improvement of antioxidant defense mechanisms from Leydig cells will require further investigation.

To better understand the mechanisms of gene expression regulation by gigantol, other polyphenols, such as ferulic acid and isoferulic acid, were compared. Gigantol, ferulic acid and isoferulic acid have a common hydroxy-methoxyphenyl moiety. Interestingly, ferulic acid reduced arsenic-induced oxidative stress, testicular damage and sperm abnormalities through regulation of the expressions of *Nfe2l2*, *Ppargc1a*, and *Star* [54]. Although *Star* expression was increased in response to treatment with ferulic acid in this study, improvement in testosterone production was non-significant. However, others reported that ferulic acid administration to male rats for 8 weeks resulted in an increased number of Leydig cells per testis and testosterone production [55]. Precisely, administration of ferulic acid to male rats with lead-induced testicular damage restored serum testosterone levels [55]. Being present in honey with ellagic acid, rosmarinic acid, and p-coumaric acid, ferulic acid was also found to improve serum testosterone levels in male rats [56]. According to our RNA-Seq data analysis, *Nfe2l2* and *Ppargc1a* were not modulated by gigantol under our experimental conditions. In addition, ferulic acid had no effect on *Star* expression and progesterone production from MA-10 Leydig cells.

In addition to inhibition of oxidative stress, phenolic compounds, such as ferulic acid, may also increase the intracellular levels of cAMP by inhibiting phosphodiesterases [57]. Hence, improved cAMP/PKA signaling in Leydig cells may promote increased androgen biosynthesis. However, our results suggest that ferulic acid and isoferulic acid are not effective in increasing progesterone production in MA-10 cells. The association of two hydroxy-methoxyphenyl moieties as found in gigantol seems to promote the expression of cholesterol biosynthesis and steroidogenesis-related genes in these cells. In addition, having hydroxy-methoxyphenyl moieties, curcumin rather decreases LH-stimulated testosterone production from rat adult Leydig cells by inhibiting the enzyme HSD17B1 [58]. Curcumin can also inhibit cAMP-induced steroidogenesis by decreasing the expressions of CYP11A1 and StAR in mouse Leydig cells [59]. Such compound is of special interest to reduce the pathogenesis of androgen-dependent prostate cancer [55].

Our observed enhanced progesterone production in response to gigantol may be attributed to the activation of gene expression related to cholesterol and steroid biosynthesis and/or metabolism. MA-10 Leydig cells represent an excellent model for studying the regulation of steroidogenesis. Indeed, the cAMP/PKA signaling pathway is functional and participates in the regulation of steroidogenesis-related genes’ expression in these cells [45]. However, progesterone is the main product of steroid biosynthesis in MA-10 Leydig cells due to deficiencies in *Cyp17a1* and *Hsd17b3* expressions [60,61].

**Figure 12:**
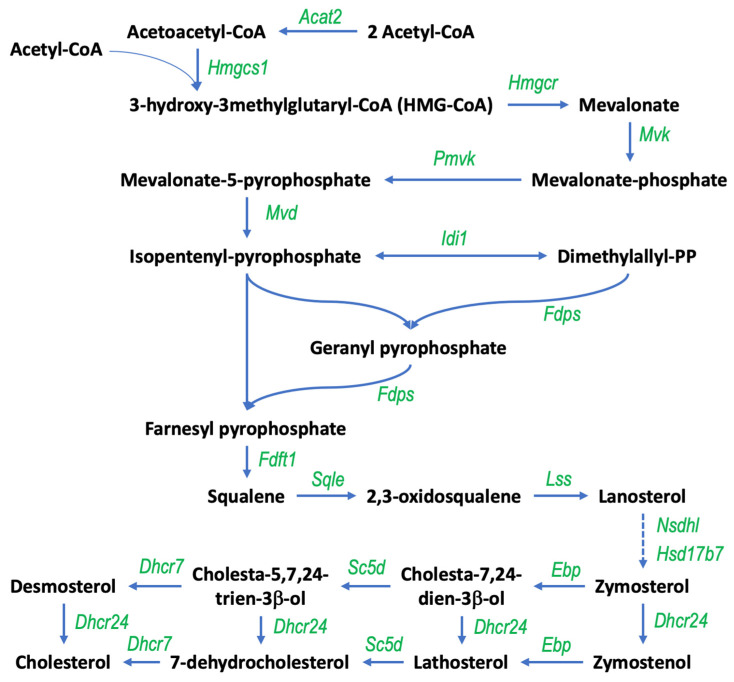
Gigantol increases the expression of genes involved in the biosynthesis of cholesterol in MA-10 Leydig cells. Uses of NADPH molecules are not shown. Abbreviations: Acat2, Acetoacetyl-CoA thiolase; Hmgcs1, HMG-CoA synthase; Hmgcr, HMG-CoA reductase; Mvk, mevalonate kinase; Pmvk, Phosphomevalonate kinase; Mvd, Mevalonate diphosphate decarboxylase; Idi1, Isopentenyl pyrophosphate isomerase; Fdps, Farnesyl pyrophosphate synthase; Fdft1, Squalene synthase; Sqle, Squalene monooxygenase; Lss, Lanosterol synthase; Nsdhl, NAD(P) dependent steroid dehydrogenase-like; Hsd17b7, Hydroxysteroid 17-beta dehydrogenase 7; Dhcr24, 24-Dehydrocholesterol reductase; Ebp, Cholestenol delta-isomerase; Sc5d, Sterol-C5-desaturase; Dhcr7, 7-Dehydrocholesterol reductase.

Cholesterol availability for steroid production depends on LDL uptake by the LDL receptor and on the rate of its synthesis. In this study, we have shown that gigantol promotes cholesterol biosynthesis and metabolism in MA-10 Leydig cells (Figure 12). Cholesterol can be synthesized de novo from acetate or from cholesterol esters in intracellular lipid droplets. There are five major stages in the process of cholesterol synthesis from acetate. Firstly, two acetyl CoA molecules are converted to 3-hydroxy-3-methylglutaryl-coenzyme A (HMG-CoA). HMG-CoA is then converted to mevalonate by the rate-limiting enzyme HMG-CoA reductase located in the cytoplasm. Mevalonate is converted to isopentenyl pyrophosphate (IPP), which is then converted to squalene, and the final step is the transformation of squalene to cholesterol. Genes coding for the enzymes catalyzing the biosynthesis of cholesterol, including mevalonate kinase (MVK), phosphomevalonate kinase (PMVK), disphosphomevalonate decarboxylase (MVD), isopentenyl pyrophosphate isomerase 1 (Idi1), lanosterol synthase (LSS), D24 sterol reductase (DHCR24), cholesterol D-isomerase (EBP), and 7-dehydrocholesterol reductase (DHCR7), were all found to be upregulated by gigantol in MA-10 Leydig cells (Figure 12). Our results suggest that newly synthesized cholesterol can be esterified and stored in lipid droplets following treatments of MA-10 Leydig cells with gigantol. This is of interest under normal physiological conditions as de novo cholesterol synthesis contributes to replenish cholesterol stores in Leydig cells [62].

In RNA-Seq experiments, the variations in gene expression in response to gigantol were less than 1.4-fold. These small changes may explain the lack of confirmation of certain target gene activations by gigantol in qPCR. On the other hand, the low activation of all the genes coding for cholesterol synthesis enzymes may have an important impact on the bioavailability of this substrate for steroidogenesis in Leydig cells.

## 5. Conclusions

Overall, gigantol increases the expression of genes involved in cholesterol and steroid biosynthesis and steroid metabolism. Although these changes in gene expression in response to gigantol are minimal, they result in an improved progesterone production in MA-10 Leydig cells. Compared to ferulic acid and isoferulic acid, the presence of two hydroxy-methoxyphenyl moieties in gigantol seems to promote its efficacy to activate gene expression related to cholesterol and steroid biosynthesis in Leydig cells. Thus, gigantol found in vegetables may contribute to an optimal production of androgens by testicular Leydig cells in males. Although gigantol shows promise in delaying the onset of male hypogonadism, this will require further investigation using aging rodent models to be confirmed.

## Figures and Tables

**Table 1:** Top positive enrichment GO terms (BP, CC, MF) of differentially expressed genes using GSEA ^1^.

Description	Gene Set	Ontology	NES	*p* Value	FDR
Steroid biosynthetic process	GO:0006694	BP	2.66	0	0
Sterol metabolic process	GO:0016125	BP	2.65	0	0
Alcohol biosynthetic process	GO:0046165	BP	2.64	0	0
Secondary alcohol metabolic process	GO:1902652	BP	2.63	0	0
Small molecule biosynthetic process	GO:0044283	BP	2.62	0	0
Organic hydroxy compound biosynthetic process	GO:1901617	BP	2.60	0	0
Cholesterol metabolic process	GO:0008203	BP	2.60	0	0
Steroid metabolic process	GO:0008202	BP	2.60	0	0
Sterol biosynthetic process	GO:0016126	BP	2.59	0	0
Isoprenoid metabolic process	GO:0006720	BP	2.56	0	0
Cytosolic ribosome	GO:0022626	CC	2.65	0	0
Ribosomal subunit	GO:0044391	CC	2.58	0	0
Cytosolic large ribosomal subunit	GO:0022625	CC	2.54	0	0
Ribosome	GO:0005840	CC	2.51	0	0
Large ribosome subunit	GO:0015934	CC	2.48	0	0
Mitochondrial matrix	GO:0005759	CC	2.37	0	0
Cytoplasmic part	GO:0044445	CC	2.30	0	0
Microbody	GO:0042579	CC	2.30	0	0
Peroxisome	GO:0005777	CC	2.29	0	0
Cytosolic small ribosomal subunit	GO:0022627	CC	2.26	0	0
Structural constituent of ribosome	GO:0003735	MF	2.57	0	0
rRNA Binding	GO:0019843	MF	2.38	0	0
Intramolecular oxidoreductase activity	GO:0016860	MF	2.18	0	0.001
Ligase activity	GO:0016874	MF	2.16	0	0.002
Lyase activity	GO:0016829	MF	2.15	0	0.002
Carbon-carbon lyase activity	GO:0016830	MF	2.14	0.001	0.002
snoRNA binding	GO:0030515	MF	2.13	0	0.003
Oxidoreductase activity, acting on the ch-ch group of donors	GO:0016627	MF	2.12	0	0.003
Oxidoreductase activity, acting on ch-oh group of donors	GO:0016614	MF	2.09	0	0.005
Isomerase activity	GO:0016853	MF	2.08	0	0.006

^1^ Abbreviations: BP, biological process; CC, cellular component; FDR, false discovery rate; GO, gene ontology; GSEA, gene set enrichment analysis; MF, molecular function; NES, normalized enrichment score.

## Data Availability

The RNA-Seq data presented in this study are available at: https://www.ncbi.nlm.nih.gov/bioproject/PRJNA783636.
